# Parotid Sialolithiasis Diagnosed on Point of Care Ultrasound (POCUS)

**DOI:** 10.24908/pocusj.v10i01.18657

**Published:** 2025-04-15

**Authors:** Heather Lystad, Elaine Yu, Rachna Subramony

**Affiliations:** 1 Department of Physical Medicine and Rehabilitation, University of North Carolina, Chapel Hill, NC, USA; 2 Department of Emergency Medicine, University of California San Diego, San Diego, CA, USA

**Keywords:** sialolith, sialolithiasis, parotid, ultrasound, POCUS

## Abstract

Sialoliths are the most common salivary gland pathology. Point of care ultrasound (POCUS) is a useful method for identifying and locating sialoliths in acutely presenting patients. POCUS can detect salivary stones with high sensitivity and accuracy and decreases the need for radiation exposure from other imaging modalities. In this case, we describe a 58-year-old woman without significant past medical history who presented to the emergency department with left-sided facial pain and swelling without infectious symptoms. A facial POCUS examination was performed on her left cheek, which identified an echogenic sialolith obstructing the parotid duct with associated ductal dilation. This allowed for prompt diagnosis without a need for further imaging.

## Case Report

A 58-year-old woman with a past medical history of irritable bowel syndrome presented to the emergency department due to gradual swelling and pain on the left side of her face for three weeks. She also reported feeling a lump on her inner cheek. The patient experienced worsened swelling, pain with opening her mouth, and shooting pains on the left side of her face. She denied any fevers or chills. She recalled a similar episode in the past for which she was evaluated but did not follow up.

**Figure 1.**
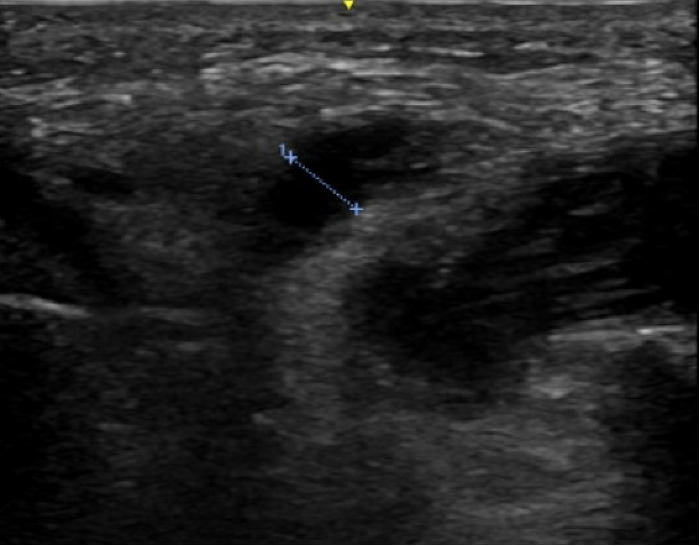
Left parotid duct (4.6 mm), transverse view using point of care ultrasound (POCUS).

**Figure 2.**
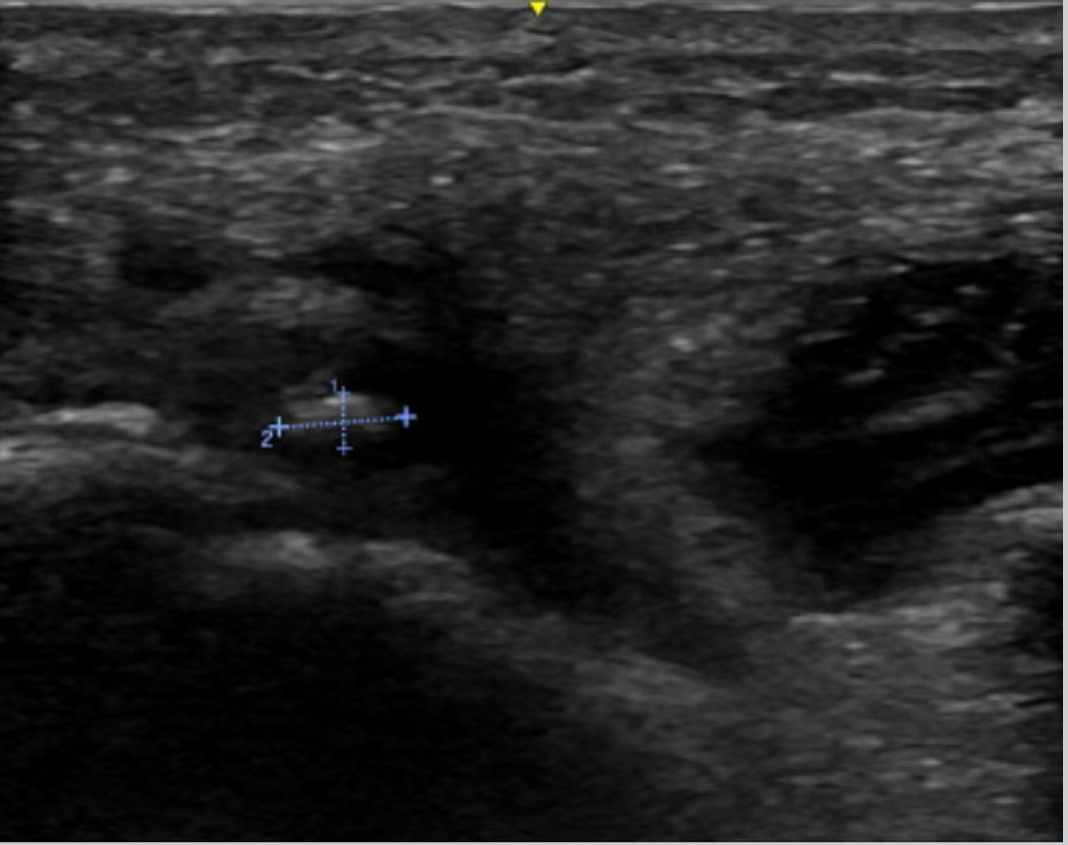
Left parotid duct sialolith (2 mm x 4.5 mm), transverse view on point of care ultrasound (POCUS) examination.

On presentation, the patient had a blood pressure of 105/67, heart rate of 57, respiratory rate of 16, temperature of 98.9F, and oxygen saturation of 96% on room air. Left cheek and facial swelling was observed on her physical exam. A hard, immobile mass was palpated on the inner aspect of her left cheek. No purulence was expressed with massage. A POCUS examination was performed to evaluate the bilateral parotid glands and ducts. The POCUS examination revealed a dilated left parotid duct (Figure 1) with echogenic obstructing stone (Figure 2, Supplement 1).

## Discussion

Sialolithiasis is the most common disorder of the salivary glands, with post-mortem analysis demonstrating sialoliths in just over 1% of the population [1]. Sialolithiasis is more common in men compared to women, and in patients over the age of fifty [2]. While the exact cause of stone formation is unknown, ductal stasis and a nidus for precipitation are presumed prerequisites. Salivary stones form when calcium phosphate and calcium carbonate, within an organic matrix of glycoproteins and mucopolysaccharides, form around a core of material such as desquamated epithelial cells, bacteria, mucus, or a foreign body [1–3]. In cases where systemic gout is the etiology, stones are made of uric acid [3].

While an estimated 80% of salivary stones originate in the submandibular gland, approximately 10-20% of sialoliths occur in the parotid gland, with sublingual and minor salivary gland stones being much less common [1]. This is thought to be caused by gravity affecting submandibular saliva flow, as well as the more viscous secretion of the submandibular gland compared to the more serous secretions of the parotid gland [2,4].

Plain radiographs may be used to detect sialoliths that are radiopaque, an attribute of 90% of submandibular stones but only 20% to 60% of parotid stones [2,5]. Saliva from the submandibular gland is relatively alkaline with higher hydroxyapatite and phosphate content when compared with parotid stones, likely contributing to this difference [2,4].

High-resolution non-contrast computed tomography (CT) with fine cuts can detect stones with high sensitivity and is the current imaging modality of choice for sialolith evaluation [6]. However, ultrasound is the preferred modality of experienced sonographers for detecting salivary stones, and exposes patients to less radiation than CT. Previous reports show the detection of sialoliths with ultrasound have a sensitivity and specificity of 94% and 100%, respectively, with 96% accuracy [5]. Additionally, ultrasound can detect most non-radiopaque stones [5].

Parotid gland ultrasound requires a high-frequency linear transducer. The parotid gland is in the retromandibular fossa and consists of a superficial and deep lobe [7]. Its main excretory duct (Stenson's duct) runs superficially along the masseter muscle before turning medially and crossing the buccinator muscle [8]. The parotid gland can be visualized wrapping around the angle of the mandible. An accessory gland may be visualized on the medial cheek and can be used to help locate Stenson's duct. The patient's head should be tilted toward the unaffected side for easier assessment. It may also be helpful for the sonographer (or the clinician performing a POCUS examination) to place a finger on the papilla on the inside of the cheek when evaluating the parotid duct [9]. Parotid glands should be compared bilaterally, as it is not uncommon for there to be multiple stones present [8].

**Figure 3.**
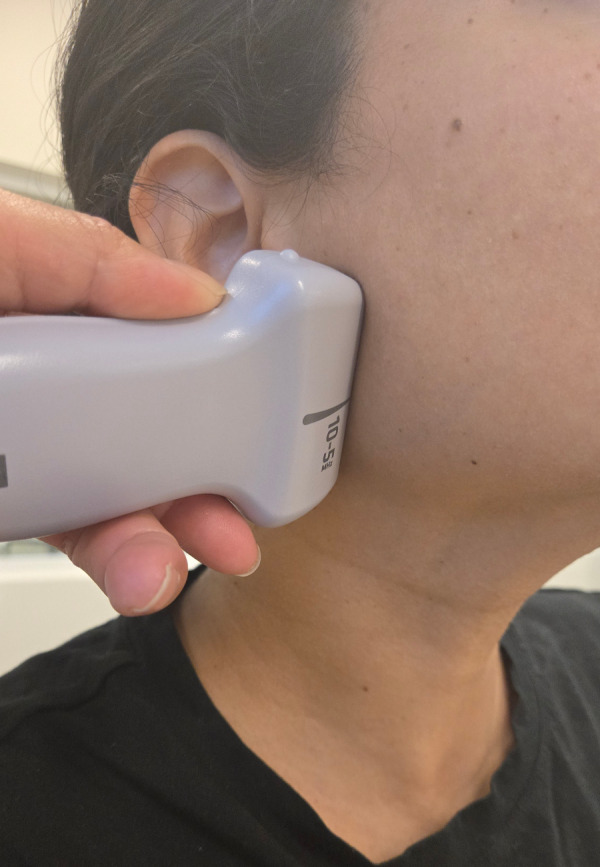
Longitudinal scanning plane of the parotid gland using point of care ultrasound (POCUS).

**Figure 4.**
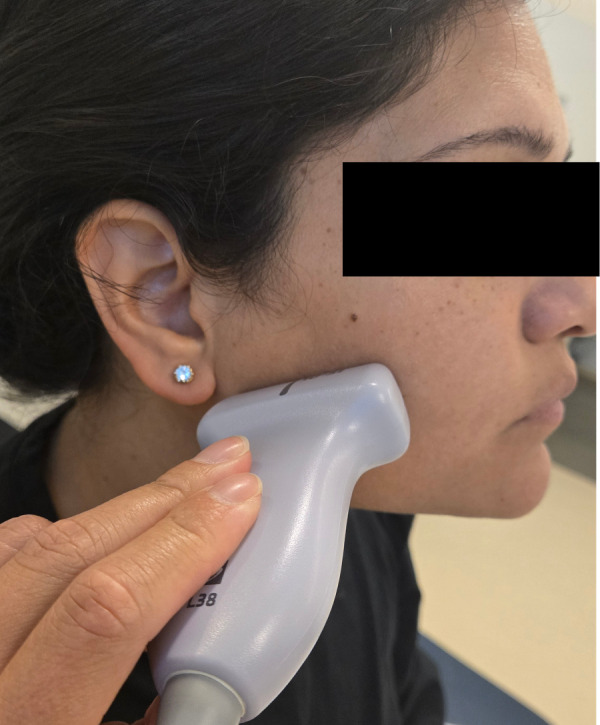
Transverse scanning plane of the parotid gland using point of care ultrasound (POCUS).

The gland should appear homogeneous and hyperechoic compared to adjacent muscles, with the degree of echogenicity depending on intraglandular fatty tissue [8]. The gland should be scanned in both the sagittal and transverse planes (Figures 3 and 4) multiple times to evaluate for size, hypervascularity, or surrounding abnormalities such as an abscess. A normal parotid duct diameter ranges from 0.1-2.3 mm [10].

As in our case, the duct proximal to the stone or acute obstruction may be visibly dilated (Figure 1). A nondilated duct is often not visualized on ultrasound or POCUS [11]. Stones appear hyperechoic due to the calcium salt content, often with distal acoustic shadowing [5]. In our case, the 2 mm x 4.5 mm stone was visualized with posterior acoustic shadowing (Figure 2). Of note, it may be difficult to visualize stones smaller than 2 mm, as they may not produce a posterior acoustic shadow [5]. However, smaller stones may still be detectable, especially with the use of sialagogues [4].

Given the diagnosis was made with POCUS, no further imaging was indicated for this patient. The patient was discharged with recommendations of massage, hydration, sialagogues, and analgesia. She was additionally scheduled to follow up with otolaryngology as an outpatient.


